# Retraction: The Downregulation of MiR-182 Is Associated with the Growth and Invasion of Osteosarcoma Cells through the Regulation of TIAM1 Expression

**DOI:** 10.1371/journal.pone.0283115

**Published:** 2023-04-21

**Authors:** The PLOS ONE Editors

The *PLOS ONE* Editors retract this article [1] because it was identified as one of a series of submissions for which we have concerns about peer review, authorship, and similarities across articles. Image similarities were noted between panels in Fig 3B in [1], between Fig 4D in [1] and Fig 5D in [2, 3], between Fig 4E in [1] and Fig 3D in [4, 5], and between Fig 5B in [1], Fig 5D in [6, 7], Fig 4A in [8, 9] and Fig 7E in [10]. These concerns call into question the validity and provenance of the reported results. We regret that the issues were not addressed prior to the article’s publication.

All authors either did not respond directly or could not be reached.
